# Hydrogeochemical and isotopic analysis for interpreting the formation of the complex geothermal system in the Guide Basin, Northeastern Tibetan Plateau

**DOI:** 10.1371/journal.pone.0317694

**Published:** 2025-02-10

**Authors:** Yude Lei, Zhen Zhao, Guangxiong Qin, Ruishou Ba, Shaokang Yang, Haoxin Shi

**Affiliations:** 1 Key Lab of Geo-Environment Qing Hai Province, Xining, China; 2 Environmental Geological Prospecting Bureau of Qinghai Province, Xining, China; 3 Qinghai 906 Engineering Survey and Design Institute Co., Ltd., Xining, China; 4 Qinghai Institute of Geology and Environment Survey, Xining, China; 5 College of Construction Engineering, Jilin University, Changchun, China; Yogi Vemana University, INDIA

## Abstract

The Guide Basin, located in the northeastern Tibetan Plateau, is rich in geothermal resources. However, whether the genesis of all geothermal waters in the basin is consistent remains an unresolved question. To clarify the geothermal system in this area, this study investigated the hydrogeochemical and isotopic characteristics of geothermal waters, combined with an analysis of the distribution and properties of regional faults. The study analyzed the processes controlling the chemical composition of thermal waters and the reservoir temperatures, ultimately creating a conceptual model of geothermal fluids. The results indicate that the geothermal waters in the Luohantang and Zhacanggou areas are classified as Na-SO_4_·Cl type, while those in the Xinjie area are classified as Na-HCO_3_ and Na-HCO_3_·Cl type. The chemical composition of geothermal waters is primarily controlled by the weathering of silicates, with some influence from carbonate dissolution and cation exchange processes. Isotope data (δD, δ^18^O, and ^87^Sr/^86^Sr) indicate that all geothermal waters originate from atmospheric precipitation and undergo deep circulation. The heat source in Guide Basin comes from mantle heat flow and granite radioactive decay, but the thermal storage patterns in the three regions of the basin are different. The use of cation and silica geothermometers estimates the reservoir temperatures in the basin to range between 82.4 °C and 229 °C. This study enhances the understanding of the genesis of geothermal resources in the northeastern Tibetan Plateau and provides important information for guiding future geothermal exploration in the area.

## 1. Introduction

With the ongoing challenges of global climate warming and energy shortages, countries are actively promoting the development of low-carbon, efficient renewable energy to facilitate sustainable environmental development. Geothermal energy, due to its clean, stable, renewable characteristics and substantial global reserves, has been developed and utilized on a large scale worldwide [1–5].

As a key country in promoting energy transition and achieving carbon neutrality goals, China has issued documents such as the “14th Five-Year Plan for National Economic and Social Development of the People’s Republic of China and the Outline of Long-Term Goals for 2035”(www.gov.cn) and the “Action Plan for Carbon Peaking Before 2030” (mohrss.gov.cn). These policies clearly state the need to increase the development and utilization of renewable energy, particularly geothermal energy. Against this backdrop, China has conducted extensive work in the exploration and development of geothermal resources [6–11], especially in the Gonghe-Guide Basin in the northeastern Tibetan Plateau, which is considered to have rich geothermal resources and significant development potential [12–15].

However, to achieve the sustainable development of geothermal resources, it is essential to gain an in-depth understanding of the geothermal system characteristics in the region [16]. Water, as a core element that sustains the Earth’s ecological systems and geological activities, plays a crucial role in this context [17].

In geology, water not only promotes rock weathering and mineral cycling [18–20] but also serves as the primary medium for material exchange and heat transfer [21,22]. As the core fluid in geothermal systems, geothermal water is not only the main carrier of thermal energy but also an important tool for revealing the structure and dynamics of geothermal systems [23]. By transporting the reaction products between underground rocks and fluids, geothermal water provides abundant information regarding heat sources, geological structures, and water circulation pathways. Its chemical and isotopic characteristics can help determine the sources of geothermal fluids, circulation depths, and the geological processes involved [24–27]. This makes geothermal water key to interpreting the complex origins of geothermal systems.

Research has already been conducted on the northeastern Tibetan Plateau. For example, Zhang et al. (2018) inferred the causative mechanisms of thermal anomalies in the Gonghe-Guide Basin using temperature data from deep geothermal wells and measurements of thermal physical properties of rock samples [28]. Rui et al. (2024) explained the formation of the complex geothermal system in the northeastern Tibetan Plateau’s Gonghe Basin through geothermal fluid chemistry and isotopes [29]. Weinert et al. (2020) assessed the thermal physical and mechanical properties of rocks in the Guide area [30]. Liu et al. (2022) analyzed the geothermal genesis in the Zhacanggou area of the Guide Basin through hydrogeochemistry and isotopes [31]. Although the above studies provide rich information on the geothermal systems in the northeastern Tibetan Plateau, comprehensively explaining the complex genesis of the Guide Basin’s geothermal system still faces many challenges. Firstly, existing research has primarily focused on the Gonghe area and the Zhacanggou area, lacking a systematic analysis of the hydrogeochemical processes in the Guide Basin. Additionally, in areas where fault zones are developed, it is difficult to accurately trace the circulation paths of geothermal water, and the mixing of waters from different sources, as well as the interactions between deep and shallow waters, add to the complexity of the study [32,33].

The main objectives of this study are: (1) to explain the genesis of the complex geothermal system in the Guide Basin through a detailed investigation of the hydrogeochemical characteristics of geothermal water; (2) to explore water-rock reactions, heat source distribution, and fluid circulation mechanisms by analyzing the chemical composition and isotopic characteristics of geothermal water; and (3) to estimate reservoir temperatures and the circulation depths of groundwater using geochemical methods, and to develop a conceptual model of geothermal fluids in the Guide Basin. The results of this research will provide a scientific basis for the assessment and development of geothermal resources in the Guide Basin and offer guidance for future sustainable geothermal energy development.

## 2. Study area

### 2.1. Topography and climate environment

**Fig 1:**
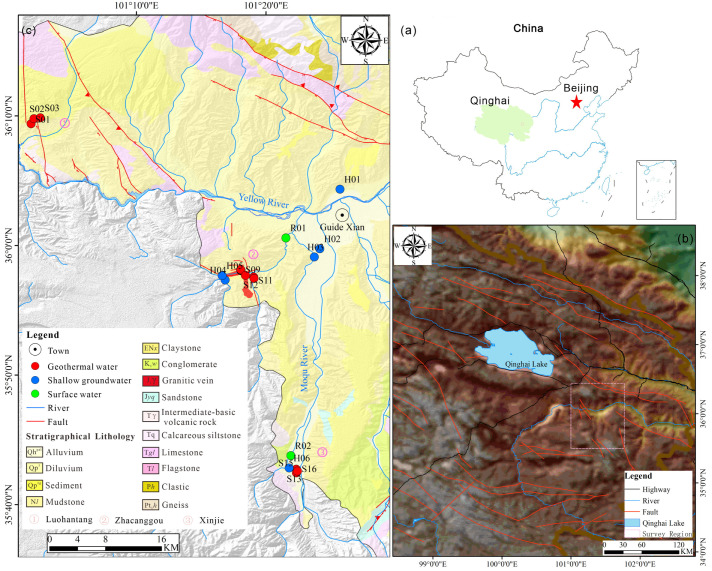
(a) Location map of the study area; (b) Simplified tectonic map of the northeastern Tibetan Plateau; (c) Simplified geological map of the Guide basins, showing sampling locations.

The Guide Basin is located in the northwestern inland region of China (Fig 1a) and features a continental climate that is arid to semi-arid, with distinct vertical climate zoning. As the altitude increases, temperatures drop while precipitation rises and evaporation decreases, leading to a climate typical of high-altitude regions, characterized by large diurnal temperature variations. The basin is surrounded by the Qinghai south Mountain, Laji Mountain, Luobuleng Mountain, Wali Pass Mountain, and Zhama Mountain, with altitudes ranging from 3500 to 4500 meters. The terrain is steep, with well-developed water systems, and the Yellow River flows from west to east through the basin, cutting to a depth of up to 900 meters, creating significant erosional landforms.

### 2.2. Geological and hydrogeological environment

Tectonically, the Guide Basin is adjacent to the Zongwulong-Qinghai Nanshan structural belt to the northeast and the Zhongqilian Laqi Mountain structural belt to the north, while to the west lies the Xinjie-Wali Pass strike-slip thrust structural subbelt. On the eastern side, the Zama Mountain strike-slip thrust structural subbelt is situated above it (Fig 1b) [34,35]. The basin’s basement is primarily composed of the Early to Middle Triassic Longwuhe Formation, the Middle Triassic Gulanggou Formation, the Late Triassic Rinaore Formation, and Indosinian-Yanshanian granites [36]. The main fault is the Xinjie-Wali Guan Fault, which runs in a NNW (north-northwest) direction and consists of several discontinuous subsidiary faults, including the Zuona Fault in the north, the Shengou Fault and Reguang Fault in the central part, and the Xinjie Fault in the south. Along both sides of the fault, Indosinian intermediate-acid intrusive rocks are widely developed, indicating that it is a deep and major fault with multiple phases of activity. The basin’s basement consists mainly of Triassic low-grade metamorphic strata and Indosinian concealed granite bodies. Regionally, the Luohantang geothermal water, Zhacanggou geothermal water, and Xinjie geothermal water are distributed in a zonal pattern along the Xinjie Fault Zone (Fig 1c).

## 3. Materials and methods

The study was conducted in May 2016 and included field surveys, on-site testing, and the collection of water samples from surface water, shallow groundwater, and geothermal water in the study area. Water samples were collected from various locations within the research area, including 2 surface water samples (R01, R02), 6 shallow groundwater samples (H01–H06), and 16 geothermal water samples (S01–S16). In order to facilitate analysis, the collected water samples were categorized into three regions based on their geographical locations: Luohantang, Zhacanggou, and Xin Jie (Fig 1c).

The on-site measurements of temperature, pH, and conductivity were carried out using a portable multi-parameter water quality analyzer (model: WTW Multi 3400i). Water samples were stored in 550 ml polyethylene bottles, which were rinsed three times with the sample water before collection. During sampling, the bottle was submerged into the sampling area to ensure no air bubbles were present inside, then sealed with a sealing film and labeled.

**Fig 2:**
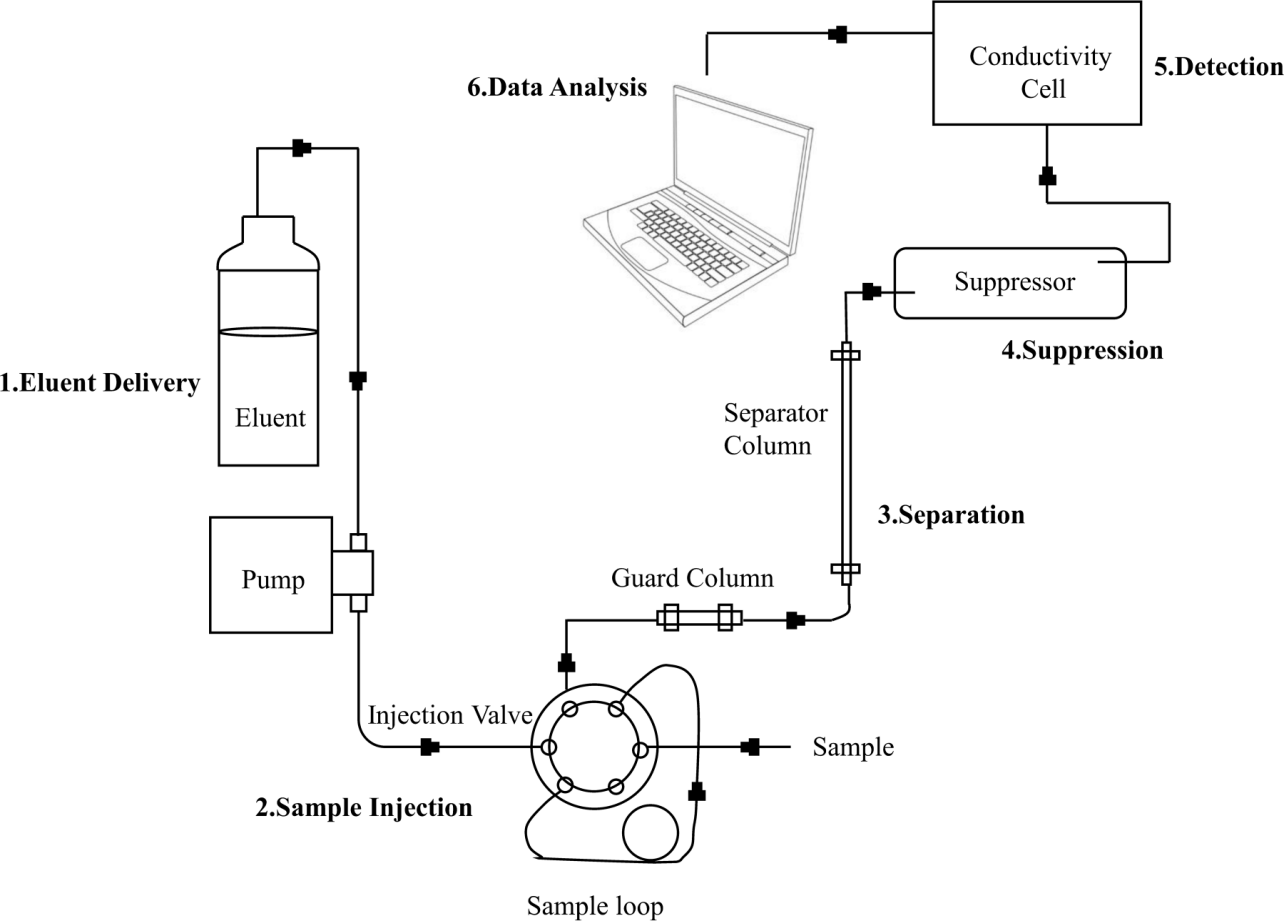
Ion analysis flowchart.

Samples intended for cation analysis were acidified with concentrated nitric acid to pH < 2. Samples for anion analysis were not acidified. The collected samples were delivered to the laboratory for testing within 72 hours. Anions and cations were determined using ion chromatography (ICS-1100, conductivity detector used in water chemistry analysis) and ICP-OES (Inductively Coupled Plasma Optical Emission Spectroscopy, ICAP-6300 is the specific model of the ICP-OES device.), respectively (Eq 1)., as shown in Fig 2. The stable isotope analyzer (LGR IWA-35-EP) measures the isotope ratios of hydrogen and oxygen in the sample and compares them with the international standard material (VSMOW, Vienna Standard Mean Ocean Water) to calculate the δD and δ^18^O values (Eq 2).

The diagram includes pumps, injection valves, column heaters and conductivity cell. Depending on the inspection needs, different types of guard column, separator column and suppressors are used. The instrument can be controlled through the LCD touch screen, and the analog output signal on the back panel can be connected to the recorder.

Ion concentration calculation formula:


$$\rho =\frac{\text{h}-h_{0}-a}{b}*f$$ (1)

δD and δ^18^O calculation formula:


$$\delta =\left(\frac{Rsample}{Rstandard}-1\right)*1000‰$$ (2)

Where *ρ* is the mass concentration of anion/cation in the sample, in mg/L; h is the peak area of anion/cation in the sample; *h*_0_ is the peak area of anion/cation in the laboratory blank sample; *a* is the intercept of the regression equation; *b* is the slope of the regression equation; *f* is the dilution factor of the sample; *δ* represents the per mil deviation of the isotope ratio in the sample relative to the international standard; *Rsample* is the ratio of (D/H) or (^18^O/^16^O) in the sample; and *Rstandard* is the ratio of (D/H) or (^18^O/^16^O) in the standard material.

## 4. Results and discussion

### 4.1. Analysis of hydrochemical characteristics

**Fig 3:**
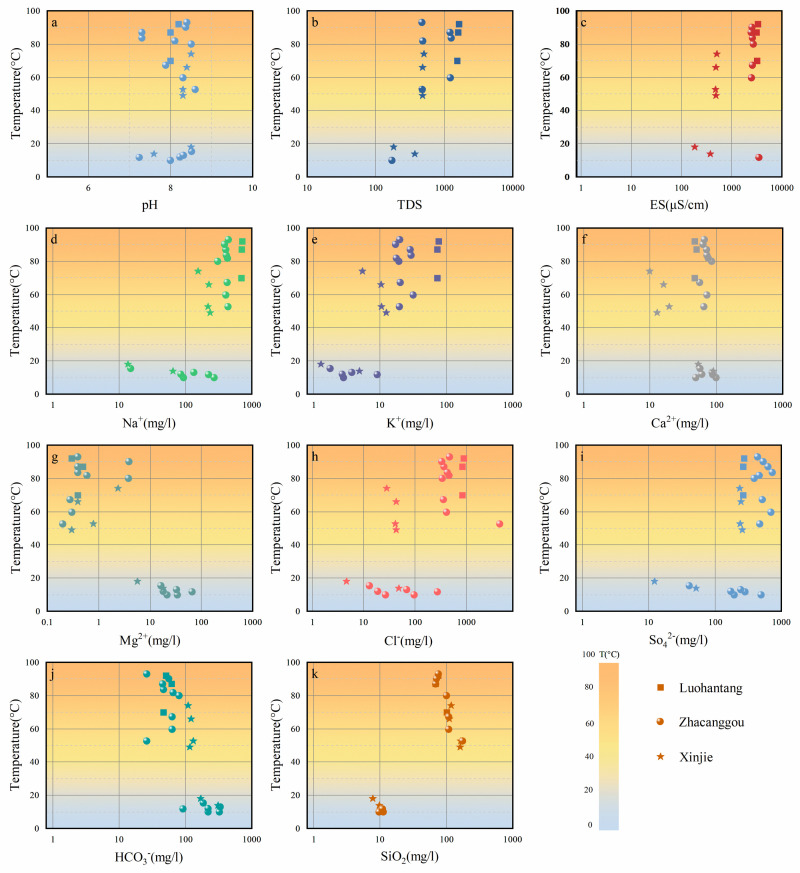
Scatter plots of main component concentrations, temperature, pH, TDS and ES in the study area.

Fig 3 shows the temperature, pH, total dissolved solids (TDS), conductivity, and hydrochemical components of water samples from the Guide Basin. Based on the “Technical Methods and Applications for Geological Environmental Monitoring” published by the China Geological Environment Monitoring Institute, the water samples are classified into geothermal water and cold water (surface water and shallow groundwater) with a boundary of 40 °C [37], Descriptive statistics of hydrochemical variables for the samples are presented in Table 1, with detailed test results provided in S1 Table. The temperature range of geothermal water is 48.9 °C to 93 °C, with an average temperature of 74.1 °C. The temperature of cold water ranges from 10 °C to 18 °C, with an average temperature of 12.99 °C. The temperature of cold water is close to the local air temperature, indicating good connectivity of shallow groundwater in the region.

**Table 1:** Descriptive statistics of hydrochemical variables in samples.

Parameter	Geothermal water			Cold water		
	Minimum	Maximum	Mean	Minimum	Maximum	Mean
T(°C)	48.9	93	74.1	10	18	12.99
PH	7.3	8.6	8.15	7.24	8.52	8.06
TDS (mg/L)	473.7	1639	914.1	172	375	243.6
ES (μs/cm)	981	3344	2337	764	3520	2142
K^+^ (mg/L)	5.5	77	29.12	1.3	9.17	3.776
Na^+^ (mg/L)	156	720.5	413.65	64.7	272.2	112.76
Ca^2+^ (mg/L)	10	85.8	51.96	48.6	98.83	72.88
Mg^2+^ (mg/L)	0.2	3.94	0.964	5.8	66.06	26.66
Cl^−^ (mg/L)	28.4	4448.7	647.2	4.7	273.43	68.9
HCO_3_^−^ (mg/L)	26.1	131	69.5	91.79	307.9	231.6
SO_4_^2−^ (mg/L)	235.4	734.2	416	12.3	494.9	186.8
CO_3_ (mg/L)	0	19.3	3.99	0	6.4	2.83
SiO_2_ (mg/L)	68.9	176.4	111.28	7.8	11.2	9.88

Typically, an increase in temperature leads to a decrease in pH [38]. However, the pH of geothermal water in the study area ranges from 7.3 to 8.6, with an average of 8.15, indicating a neutral to weakly alkaline nature. This alkalinity can be attributed to factors such as mineral dissolution and water-rock interaction within the geothermal system, which increase the concentration of OH^−^ ions in the water [39].

The TDS of geothermal water ranges from 473.7 mg/L to 1639 mg/L, with an average of 914.1 mg/L, significantly higher than that of cold water (172 mg/L to 375 mg/L, with an average of 243.6 mg/L). The higher TDS values in geothermal water reflect longer circulation and residence times within the aquifer, as well as more intense geothermal dissolution processes [40]. The electrical conductivity (EC) of geothermal water varies: it ranges from 2465 μS/cm to 3344 μS/cm in the Zhacanggou and Luohantang areas, while in the Xinjie area, it ranges from 980 μS/cm to 996 μS/cm, which is only 20% higher than that of cold water. This suggests that the circulation depth in this region may be relatively shallow or that there is a certain proportion of mixing between geothermal water and cold water.

**Fig 4:**
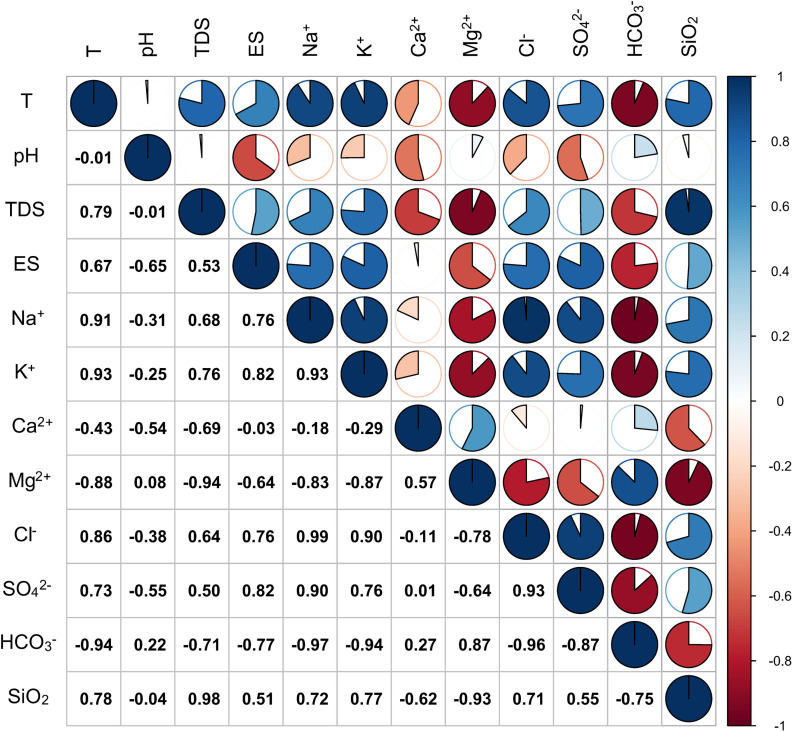
Correlation diagram of main components and temperature in the study area.

**Fig 5:**
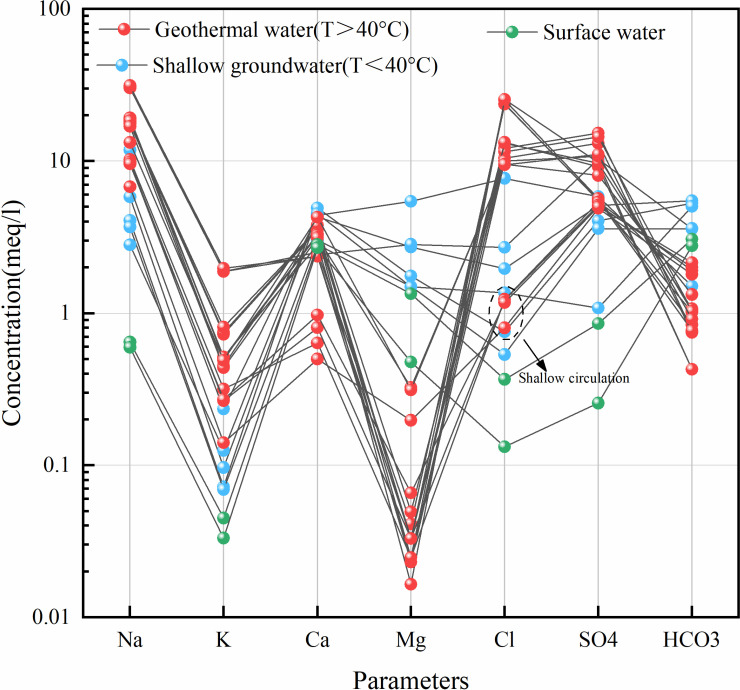
Semi-logarithmic schoeller diagram showing the distribution of geothermal water, shallow groundwater, and surface water in the study area.

**Fig 6:**
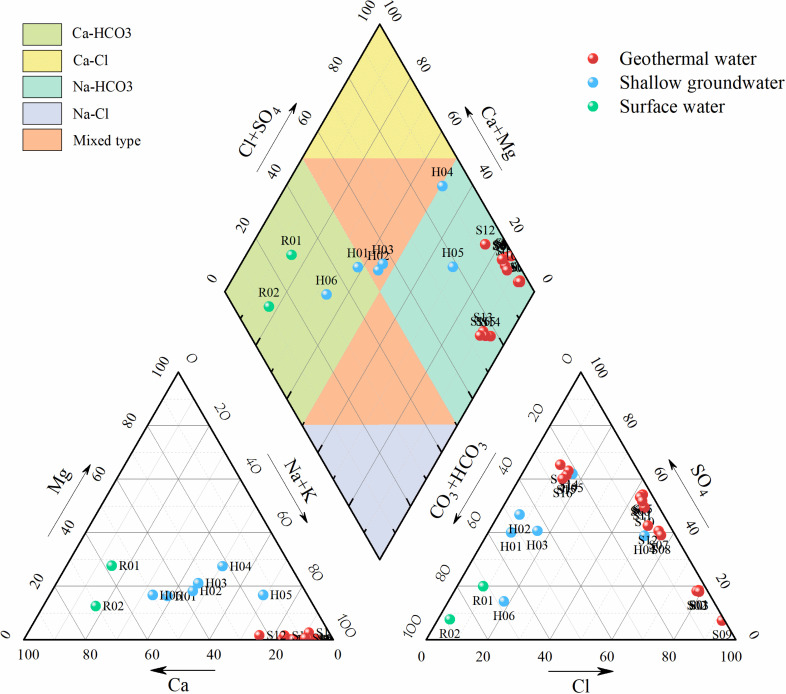
Piper diagram of all the water samples in the study area.

The hydrochemical type of groundwater is an important means to reveal the groundwater geochemical environment. Fig 3 shows the distribution relationship between sampling temperature and water chemical parameters, and Fig 4 analyzes the correlation between each parameter. From the first column of results, it can be seen that temperature is positively correlated with TDS, ES, Na^+^, K^+^, Cl^−^, SO_4_^2−^ and SiO_2_, and negatively correlated with Ca^2+^, Mg^2+^ and HCO_3_^−^, with little correlation with pH. With the increase of temperature, the concentrations of TDS, ES, Na^+^, K^+^, Cl^−^, SO_4_^2−^ and SiO_2_ increased, while the concentrations of Ca^2+^, Mg^2+^ and HCO_3_^-^ were relatively low. To better understand the hydrochemical characteristics and their causes in the study area, the schoeller classification method was used to categorize the hydrochemical types of different water bodies. Based on the hydrochemical data (Na^+^, K^+^, Ca²^+^, Mg²^+^, Cl^−^, SO_4_²^−^, HCO_3_^−^) in milliequivalents, semi-logarithmic schoeller diagrams (Fig 5) and Piper trilinear diagrams (Fig 6) were created.

From the semi-logarithmic schoeller diagram, it can be observed that the concentrations of Na^+^, K^+^, Cl^−^, and SO_4_²^−^ in geothermal water are generally higher than those in shallow groundwater and surface water. This suggests that geothermal water has reacted with Na^+^, K^+^-rich feldspar minerals and soluble salts such as Cl^−^ and SO_4_²^−^ [41]. In contrast, the concentrations of Ca²^+^, Mg²^+^, and HCO_3_^−^ are relatively lower, likely because high temperatures promote the dissolution or precipitation of carbonate minerals, thereby reducing the concentrations of these ions [42]. The lower Cl^−^ concentration in the geothermal water of the Xinjie area indicates that different environmental conditions may have existed during the formation of geothermal water compared to the Luohantang and Zhacanggou areas.

In the cation triangle of the Piper diagram, Na^+^ is the predominant cation in geothermal water (Fig 6), while the cations in shallow groundwater and surface water are primarily composed of Na^+^ and Ca²^+^. The anion triangle shows a more scattered distribution of anions, with the main anions in geothermal water being SO_4_²^−^, Cl^−^, and HCO_3_^−^, whereas shallow groundwater and surface water are primarily dominated by SO_4_²^−^ and HCO_3_^−^.

1. In the Luohantang and Zhacanggou areas (S1–S12), the water samples exhibit high concentrations of Na^+^, SO_4_²^−^, and Cl^−^, while the concentrations of Mg²^+^ and HCO_3_^−^ are relatively low, indicating that these waters belong to the Na-SO_4_·Cl type. The chemical composition of the water in the study area is significantly influenced by fault activity, lithology, and groundwater circulation [43]. The geological complexity of the Zhacanggou area, characterized by a high content of thick granites and granodiorites formed during the Indosinian period, leads to increased concentrations of Na^+^ and K^+^. Under high-temperature and high-pressure conditions, the hydrolysis of aluminosilicate minerals by groundwater further enriches the Na^+^ and K^+^ concentrations in the water.
2. In the Xinjie area (S13–S16), the main ions are Na^+^, Cl^−^, and HCO_3_^−^, indicating that the water types are Na-HCO_3_ and Na-HCO_3_·Cl. The variation in ion concentrations is relatively small, suggesting that the flow path of the geothermal water is short [44]. Additionally, the higher concentration of HCO_3_^−^ in the water is attributed to the mixing of underground cold water.
3. The surface water in the study area mainly exhibits a Ca-HCO_3_ type, while the shallow groundwater is characterized as Na·Ca-HCO_3_·SO_4_ type.

### 4.2. Stable isotope characterization

#### 4.2.1. δ^18^O and δD isotope characterization.

**Fig 7:**
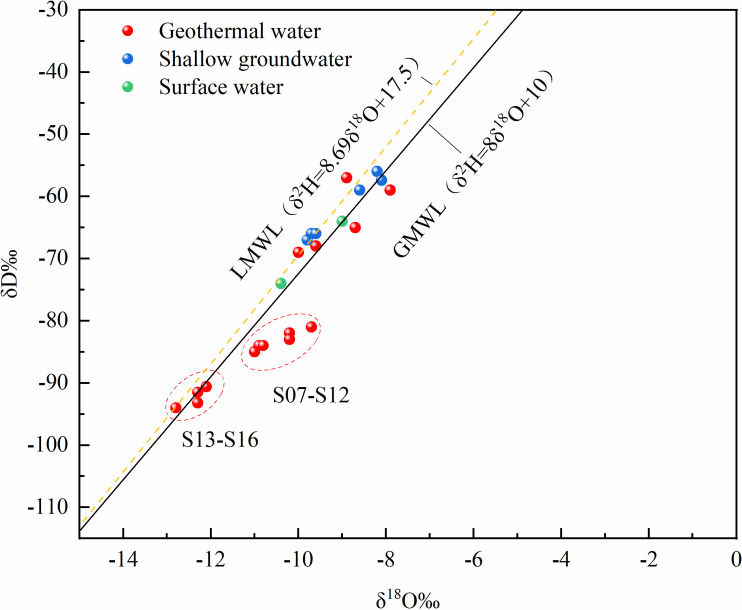
δ^18^O and δD Isotope characteristics in the study area.

Stable hydrogen and oxygen isotopes are often used as tracers in water samples to identify the source of geothermal water and the hydrological processes occurring along the flow path [45]. In this study, measurements of hydrogen and oxygen isotopes from collected water samples were conducted (Table 2), resulting in a stable isotope plot for the study area (Fig 7). The sampling points for both geothermal water and cold water are close to the Global Meteoric Water Line (GMWL) and the Local Meteoric Water Line (LMWL), indicating that the primary recharge source in this area is atmospheric precipitation [46,47].

**Table 2:** δ^18^O and δD isotope composition of water samples.

Sample ID	δD-VSMOW (‰)	δ^18^O-VSMOW (‰)
H01	−66	−9.7
H02	−66	−9.6
H03	−56	−8.2
H04	−67	−9.8
H05	−59	−8.6
H06	−57.4	−8.1
R01	−74	−10.4
R02	−64	−9
S01	−57	−8.9
S02	−65	−8.7
S03	−59	−7.9
S04	−68	−9.6
S05	−69	−10
S06	NA	NA
S07	−81	−9.7
S08	−82	−10.2
S09	−83	−10.2
S10	−84	−10.8
S11	−85	−11
S12	−84	−10.9
S13	−94	−12.8
S14	−93.2	−12.3
S15	−91.5	−12.3
S16	−90.6	−12.1

The stable hydrogen and oxygen isotope analysis results of the water samples show that δ^18^O-VSMOW ranges from −12.8‰ to −7.9‰, and the deuterium stable isotope δD-VSMOW ranges from −94.0‰ to −56‰. The isotopes vary across the three regions, and the specific analysis is as follows:

1. In the Luohantang area (S01–S03), the isotopic composition of deuterium (δD) and oxygen (δ^18^O) in geothermal water is close to the global atmospheric water line or the local atmospheric water line, resembling that of surface water and shallow groundwater. This indicates that the main recharge source of the geothermal water is likely atmospheric precipitation, with a relatively short water circulation time and no significant water-rock interaction. Alternatively, the geothermal water may have a strong hydraulic connection with local groundwater or surface water during its ascent from deeper layers to the surface [48].
2. In the Zhacanggou area (S07–S12), the hydrogen and oxygen isotopic characteristics are located below the global atmospheric water line and the local atmospheric water line, indicating an oxygen isotope shift. This phenomenon may result from a longer water-rock interaction time for the geothermal water underground, involving oxygen isotope exchange with surrounding carbonate or silicate rocks that are rich in δ^18^O [49]. This suggests that the water sources not only come from atmospheric precipitation or evaporation processes, but also from precipitation in high-altitude areas or deep closed systems [50].
3. In the Xinjie area (S13–S16), the hydrogen and oxygen isotopic characteristics are close to the global atmospheric water line (GMWL) and the local atmospheric water line, indicating that the main source of recharge for the geothermal water is atmospheric precipitation. Combined with the lower electrical conductivity and Cl^−^ content in this area, it suggests that the geothermal water may have some mixing with shallow water. However, the δD values are significantly lower than those of local cold water, further indicating that the source of the geothermal water may come from precipitation in high-altitude areas. After infiltrating underground through geological structural belts, this precipitation migrates to the study area, and during the circulation process, there is no significant water-rock interaction, which maintains the lower δD values [51].

#### 4.2.2. Sr Isotope characterization.

To further investigate the degree of interaction between groundwater and rocks in the Guide Basin, the team collected seven sets of water samples from the study area in June 2022 to analyze the ^87^Sr/^86^Sr values (Table 3).

**Table 3:** ^87^Sr/^86^Sr Isotope test results for water samples.

Number	Sample ID	Type	Temperature(°C)	pH	^87^Sr/^86^Sr	^87^Sr/^86^S rerrors	D(^87^Sr/^86^Sr)‰
1	Q01	well	90.0	8.50	0.714297	0.000010	4.297
2	Q02	well	90.2	8.37	0.714349	0.000011	4.349
3	Q03	spring	67.2	7.88	0.714336	0.000014	4.336
4	Q05	spring	11.6	7.24	0.710923	0.000012	0.923
5	Q06	spring	13.7	8.68	0.711412	0.000016	1.412
6	Q07	surface water	25.3	8.44	0.711036	0.000010	1.036
7	Q08	spring	12.4	8.08	0.711387	0.000016	1.387

**Fig 8:**
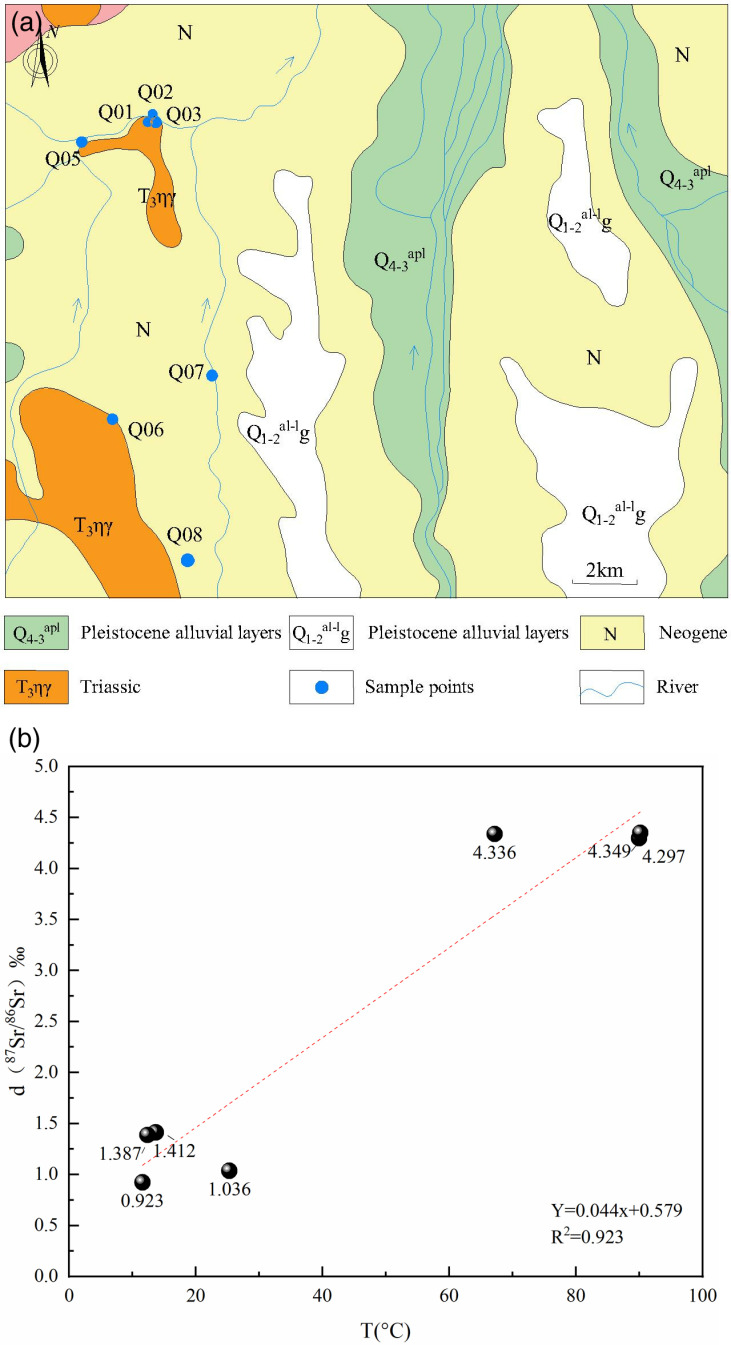
Sr isotope sampling points in the study area and variation of d(^87^Sr/^86^Sr) ratio with temperature.

The analysis results indicate that the groundwater in the area is of crustal origin, with ^87^Sr/^86^Sr values all greater than 0.710, and δ(^87^Sr/^86^Sr) values ranging from 0.923 to 4.349. Fig 8 shows that as the water sample temperature increases, the δ(^87^Sr/^86^Sr) values also gradually rise, suggesting that higher temperatures facilitate water-rock interactions. Elevated temperatures enable the accumulation of ^87^Sr isotopes in groundwater more readily. The ^87^Sr/^86^Sr values of geothermal water are significantly higher than those of cold spring water and surface water in the plains, indicating that thermal water and spring water have undergone longer water-rock interactions during their journey from recharge to discharge areas. In contrast, cold spring water and surface water in the foothill plains are mainly recharged by atmospheric precipitation, with weaker water-rock interactions and less contact with parent rocks containing Sr isotopes [52].

**Fig 9:**
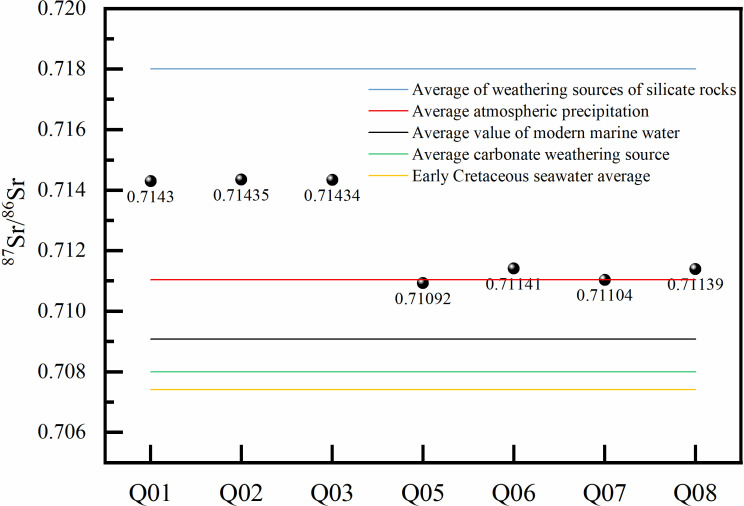
87Sr/86Sr ratios of Sr isotopes from different sources in the study area.

Meanwhile, the ^87^Sr/^86^Sr ratios of groundwater samples in the area are situated between the ratios of atmospheric precipitation and those from silicate rock weathering sources (Fig 9) [53]. The ^87^Sr/^86^Sr ratios of surface water and shallow groundwater (Q05, Q06, Q07, Q08) are very close to that of atmospheric precipitation and are significantly higher than the average ratio from carbonate rock weathering sources. This indicates that the surface water and shallow groundwater in the Guide Basin are primarily recharged by atmospheric precipitation.

The ^87^Sr/^86^Sr ratios of geothermal water (Q01, Q02, Q03) are higher than that of atmospheric precipitation but significantly lower than the ratios from silicate rock weathering sources. This indicates that deep underground geothermal water has experienced infiltration recharge from atmospheric precipitation in the surrounding mountainous areas of the basin. After undergoing deep circulation and heating, it emerges at favorable tectonic locations or is revealed through drilling. The long-term interaction between groundwater and rocks has resulted in the dissolution of rock minerals with higher ^87^Sr/^86^Sr ratios during the runoff cycle, leading to changes in the strontium isotope characteristics of the groundwater and a deviation from the ^87^Sr/^86^Sr ratio of atmospheric precipitation.

### 4.3. Major hydrogeochemical processes

During the circulation of geothermal water, Cl^−^ is almost unaffected by water-rock interactions, while ions such as Na^+^, K^+^, Ca²^+^, and Mg²^+^ are influenced by temperature and engage in water-rock interactions. Therefore, the analysis of solute molar ratios can further reveal these geochemical processes.

**Fig 10:**
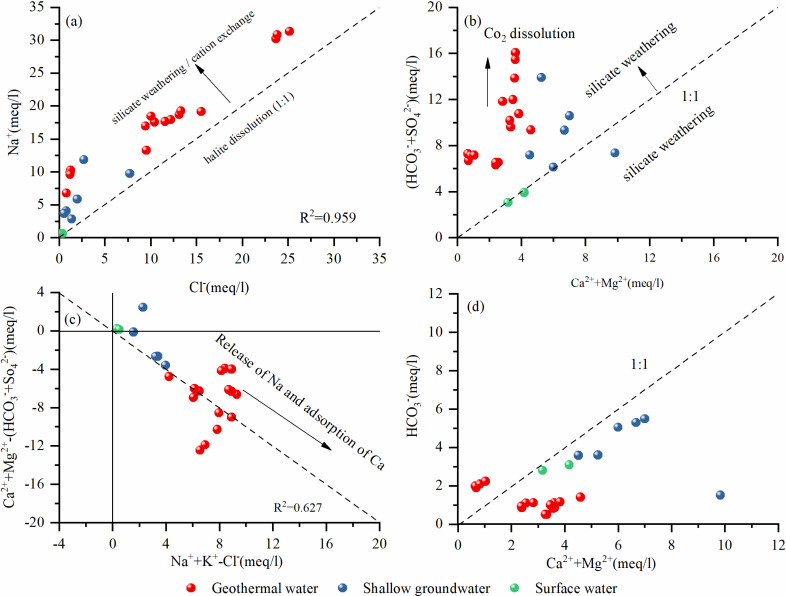
(a) Na^+^ vs Cl^−^ (b) HCO_3_^−^ + SO_4_^2−^ vs Ca²^+^ + Mg²^+^ (c) Ca²^+^ + Mg²^+^ − (HCO_3_^−^ + SO_4_²^−^) vs Na^+^ + K^+^ − Cl^−^ and (d) HCO_3_^−^ vs Ca²^+^ + Mg²^+^ for the water samples in the study area.

In the study area, geothermal water primarily contains Na^+^ as the main cation, with Cl^−^ and SO_4_²^−^ as the main anions. The high correlation between Na^+^ and Cl^−^ (r = 0.96) suggests that the geothermal fluid may be influenced by certain underlying factors. Generally, when the molar ratio of Na^+^ to Cl^−^ in groundwater is 1:1, it indicates that these ions originate from the dissolution of rock salt [54]. However, in the study area, the Na^+^/Cl^−^ molar ratio deviates from the 1:1 line (Fig 10a), suggesting that silicate weathering may be involved (Eq 3).

When the ratio of (Ca²^+^ + Mg²^+^) to (HCO_3_^−^ + SO_4_²^−^) in groundwater is 1, it indicates that Ca²^+^ and Mg²^+^ primarily originate from the dissolution of carbonates and silicates. However, Fig 10b shows that all geothermal water samples have this ratio above the 1:1 line. This suggests that cation exchange reactions may have occurred during water flow, where Ca²^+^ and Mg²^+^ are replaced by Na^+^ from the surrounding formations, leading to an increase in Na^+^ and a decrease in Ca²^+^ and Mg²^+^ in the groundwater (Eq 4 and Eq 5). The results from Fig 10a,b support the hypothesis of silicate weathering occurring in the area [55,56].

The ratio of (Ca²^+^ + Mg²^+^) to HCO_3_^−^ being 1 typically indicates that Ca²^+^ and Mg²^+^ in the water mainly originate from the dissolution of carbonate minerals. In Fig 10d, this ratio falls below the 1:1 line. Compared to the analysis in Fig 10b, this relationship suggests that after the release of calcium and magnesium and the dissolution of carbonates, the influence of sulfate on the overall ionic balance in the water is relatively minor (Eq 6). Therefore, the chemical characteristics of the water are also controlled by the dissolution of carbonate minerals.

Moreover, Fig 10c shows that geothermal water falls along the line where the slope of Ca²^+^ + Mg²^+^ − (HCO_3_^−^ + SO_4_²^−^) to Na^+^ + K^+^ − Cl^−^ is −1, but with a low correlation (r = −0.63). This indicates that the chemical composition of geothermal water is influenced not only by cation exchange reactions but also by the interplay of multiple processes [57].

In summary, the geothermal water in the study area is primarily controlled by silicate weathering, while carbonate dissolution and cation exchange processes also play a significant role.


$$2{\text{NaAlSi}}_{3}{\text{O}}_{8}+2{\text{CO}}_{2}+3{\text{H}}_{2}\text{O}\to 2\text{Na}+2{\text{HCO}}_{3}^{-}+4{\text{SiO}}_{2}$$ (3)

$${\text{Ca}}^{2+}+2\text{NaX}\to 2{\text{Na}}^{+}+{\text{CaX}}_{2}$$ (4)

$$Mg^{2+}+2\text{NaX}\to 2{\text{Na}}^{+}+{\text{MgX}}_{2}$$ (5)

$${\text{CaCO}}_{3}+{\text{H}}_{2}\text{O}+{\text{CO}}_{2}\to {\text{Ca}}^{2+}+2{\text{HCO}}_{3}^{-}$$ (6)

### 4.4. Estimation of geothermal reservoir temperature

The geothermal fluid in the reservoir usually cools significantly or mixes with cooler groundwater before reaching the surface. Therefore, the fluid temperature in the reservoir is typically higher than the surface temperature [58]. Based on the existing thermometric data and related formulas, we are able to estimate the reservoir temperature under known conditions. [59,60]. Before calculating the reservoir temperature, it is necessary to use the Na-K-Mg ternary diagram to determine the water-rock equilibrium state [61]. This diagram categorizes groundwater into three types based on its equilibrium state: fully equilibrated water, partially equilibrated water, and immature water, and it has been widely applied [29,62,63].

**Fig 11:**
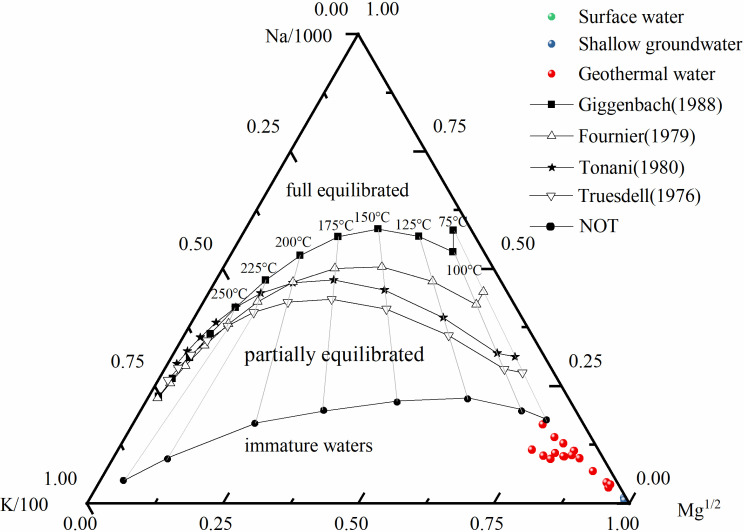
The Na-K-Mg^1/2^ triangular diagram for the water samples in the study area.

The Na-K-Mg content of the samples from the study area is plotted on the ternary diagram, as shown in Fig 11. From the figure, it can be observed that all samples fall within the immature and partially equilibrated regions, indicating that these geothermal waters have not yet reached complete chemical equilibrium. In this case, using cation geothermometers to estimate the geothermal reservoir temperature may involve some error, necessitating cautious interpretation. Additionally, the samples show a trend along the Na/K line, indicating a good linear relationship in that direction. Since silica geothermometers are less influenced by chemical equilibrium, it is advisable to consider using silica geothermometers in conjunction with cation geothermometers for a more accurate estimation of geothermal reservoir temperature. Key formulas for the geothermometer methods are presented in Table 4.

**Table 4:** Equations of Na/K and SiO_2_ thermometers.

Geothermometers	Equation (units: ° *C*)	Reference
Na/K^1^	$T=\frac{856}{0.857+\text{lg}\left(\text{Na}/\text{K}\right)}-273.15$	Truesdell(1976)
Na/K^2^	$T=\frac{1217}{1.438+\text{lg}\left(\text{Na}/\text{K}\right)}-273.15$	Fournier(1979)
Na/K^3^	$T=\frac{833}{0.780+\text{lg}\left(\text{Na}/\text{K}\right)}-273.15$	Tonani(1980)
Na/K^4^	$T=\frac{933}{0.993+\text{lg}\left(\text{Na}/\text{K}\right)}-273.15$	S. Arnórsson (1983)
Na/K^5^	$T=\frac{1319}{1.699+\text{lg}\left(\text{Na}/\text{K}\right)}-273.15$	S. Arnórsson (1983)
K/Mg^1^	$T=\frac{2330}{7.35+\text{lg}\left(K^{2}/\text{Mg}\right)}-273.15$	Fournier(1991)
K/Mg^2^	$T=\frac{1077}{4.033+\text{lg}\left(K^{2}/\text{Mg}\right)}-273.15$	Fournier(1991)
Na-K-Ca	$T=\frac{1647}{\mathrm{lg}\left(\frac{Na}{K}\right)+\beta \mathrm{lg}\left(\frac{\sqrt{Ca}}{Na}\right)+2.24}-273.15$	Fournie(1973)
SiO_2_^1^	$T=\frac{1390}{5.19-\text{lg}\left({\text{SiO}}_{2}\right)}-273.15$	Fournier (1977)
SiO_2_^2^	$T=-44.119+0.24469*{\text{SiO}}_{2}-1.7414*10^{-4}+79.305*\text{lg}\left({\text{SiO}}_{2}\right)$	Verma and Santoyo(1997)
SiO_2_^3^	$T=\frac{1032}{4.69-\text{lg}\left({\text{SiO}}_{2}\right)}-273.15$	Fournier (1977)
SiO_2_^4^	$T=\frac{1263}{5.32-\text{lg}\left({\text{SiO}}_{2}\right)}-273.15$	

Cation geothermometers are based on the principle that the equilibrium constant of cation exchange reactions changes with temperature. Commonly used geothermometers include the Na-K, K-Mg, and Na-K-Ca geothermometers [64]. The Na-K geothermometer is suitable for high-temperature geothermal waters (T > 150 °C) and has the advantage of being less affected by dilution and steam separation. The Na-K-Ca geothermometer is suitable for medium to low-temperature waters or non-equilibrium waters, while the K-Mg geothermometer is mainly used for medium to low-temperature geothermal waters.

**Fig 12:**
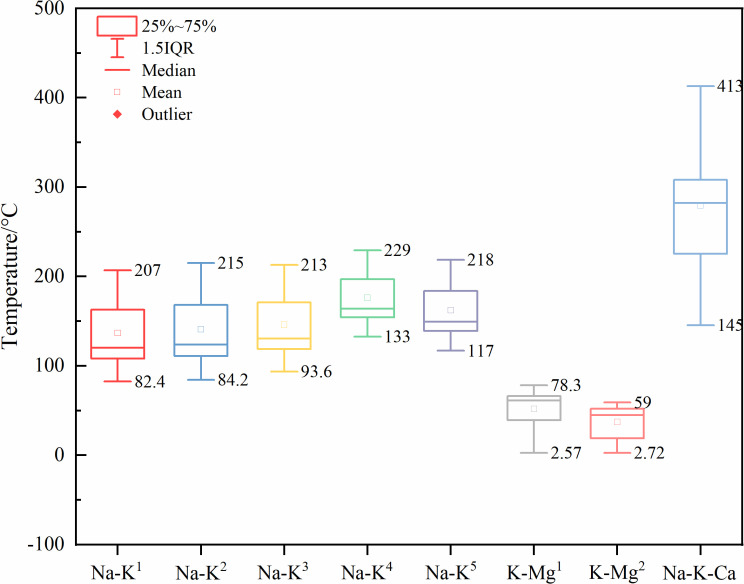
Box plots of cationic geothermometer prediction results.

When comparing the results of different cation geothermometers with the measured temperatures in the study area (ZR1: 151 °C, ZR2: 214 °C) (Fig 12) [65], it was found that the Na-K-Ca geothermometer provided abnormally high temperatures (145 °C ~ 413 °C); the K-Mg geothermometer indicated lower temperatures (2.57 °C ~ 78.3 °C, 2.72 °C ~ 59 °C); whereas the Na-K geothermometer predicted temperatures that were relatively accurate (82.4 °C ~ 207 °C, 84.2 °C ~ 215 °C, 93.6 °C ~ 213 °C, 133 °C ~ 229 °C, 117 °C ~ 218 °C).

**Fig 13:**
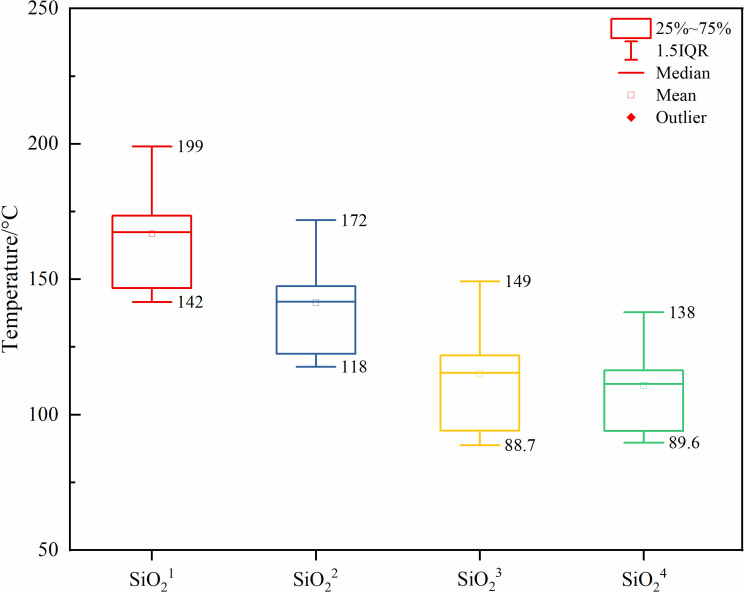
Box plots of SiO_2_ geothermometer prediction results.

The silica geothermometer estimates temperature based on the solubility of silicate minerals, with the advantages of a wide applicable temperature range and high accuracy [63]. The temperature estimates using the steam loss-free geothermometer, the improved SiO₂ geothermometer, the chalcedony geothermometer, and the calcite geothermometer are 142 °C ~ 199 °C, 118 °C ~ 172 °C, 88.7 °C ~ 149 °C, and 89.6 °C ~ 138 °C, respectively (Fig 13). Compared to the cation geothermometers, the SiO_2_ geothermometer predicts a temperature range of 88.7 °C ~ 199 °C.

Therefore, based on the results from the cation geothermometers and the silica geothermometers, the geothermal reservoir temperature is defined as 82.4 °C ~ 229 °C.

### 4.5. Groundwater circulation depth

Based on the formula for the depth of underground Geothermal water circulation:


$$Z=\frac{T_{Z}-T_{0}}{G_{g}}+Z_{0}$$ (7)

where *Z* is the depth of groundwater circulation (m), *Z*_0_ is the thickness of the thermostatic zone below the surface of the study area (m), *T*_*Z*_ is the temperature of the geothermal reservoir (°C), *T*_0_ is the average annual temperature of the study area (°C), and *G*_*g*_ is the local geothermal temperature gradient (°C/m).

The thickness of the thermostatic zone in the zone is 20 m, the local average temperature is 7.1 °C [66], and the geothermal gradient is based on the measured data from the drilled wells 41.3 ± 2.9 (°C/km) [67]. The depth of geothermal water circulation was calculated to be about 2001.6 m ~ 5859.5 m. Borehole ZR1, located in the region, had a temperature of 151.5 °C at a depth of 3000 m [68]. It can be inferred that the reservoir temperature at the depth of 5859.5 m is about 229 °C ~ 264 °C.

### 4.6. Conceptual model of geothermal water in Guide Basin

Based on multi-source data and comprehensive analytical methods, a geothermal conceptual model for the Guide Basin has been proposed. The construction of the conceptual model follows these methods:

#### Geological and Structural Analysis.

**Fig 14:**
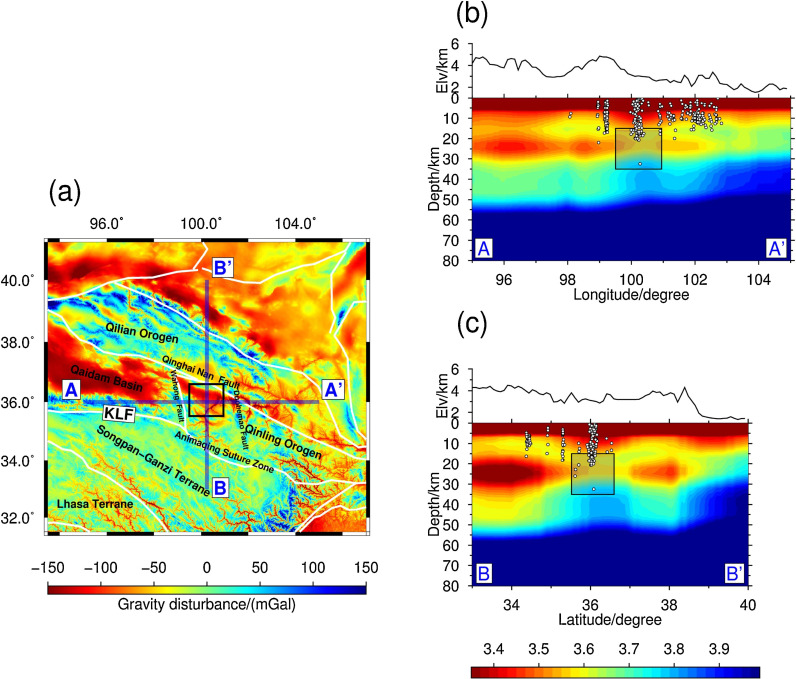
(a) Distribution of gravity anomalies in the northeastern Tibetan Plateau. The black box indicates the study area. (b) Cross section along the profile AA’ through the Vs model from ambient noise tomography. (c) Cross section along the profile through the Vs model from ambient noise tomography. The shaded boxes roughly delineate the low resistivity zones [73].

Geophysical methods such as geological surveys, gravity and magnetic anomaly data, and seismic exploration data have been used to study the geological structure of the Guide Basin. A regional geological map was created through field surveys, identifying the main fault in the area as the Xinjie-Wariguan strike-slip reverse fault, which controls heat conduction in the study area [69,70]. In the absence of drilling data or other direct observation methods, gravity anomalies serve as an important tool to reveal subsurface structural features. Gravity anomalies are often closely related to subsurface density heterogeneity and can reflect the presence of faults, folds, and other geological structures [71,72]. Wenna Zhou and Qiang Li (2023) used EIGEN-6C4 satellite gravity and aeromagnetic data to propose the crustal structure of the entire basin. At depths between 15 km and 35 km, low-density anomalies are primarily distributed in the Wahu Mountain fault zone, Xinjie fault, and the multi-river Mao fault zone on the eastern side, with minor differences in high and low-density anomalies. The Xinjie fault aligns well with known hotspots, suggesting that the geothermal energy in this region is primarily concentrated in these low-density zones and their surrounding areas [44]. Additionally, geomagnetic studies indicate that there are multiple vertical and narrow conductive zones between the near-surface and mid-crustal conductive anomalies in the Guide Basin, suggesting the presence of concealed faults within the basin. These faults may serve as pathways for heat transfer from the crustal heat sources to shallower layers (Fig 14) [73].

#### Heat Source and Heat Conduction Mechanism Analysis.

Furthermore, various methods, including borehole logging data, rock thermophysical properties, regional geophysical data, and gravity-magnetic techniques, confirm that the heat source in the study area primarily originates from the Earth’s internal mantle heat and the heat generated by the decay of radioactive elements in the crustal rocks [44,74–77].

#### Hydrological and Fluid Flow Path Analysis.

By combining stable isotope data and regional hydrological data, the recharge sources and flow paths of the geothermal water within the basin were analyzed. The study indicates that the primary recharge sources for geothermal water are atmospheric precipitation, snowmelt, and a mixture of magmatic fluids. Regional faults provide preferential infiltration pathways for geothermal water, playing a critical role in the hydrological cycle and providing the necessary dynamics and pathways for fluid movement.

#### Conceptual Model Construction.

**Fig 15:**
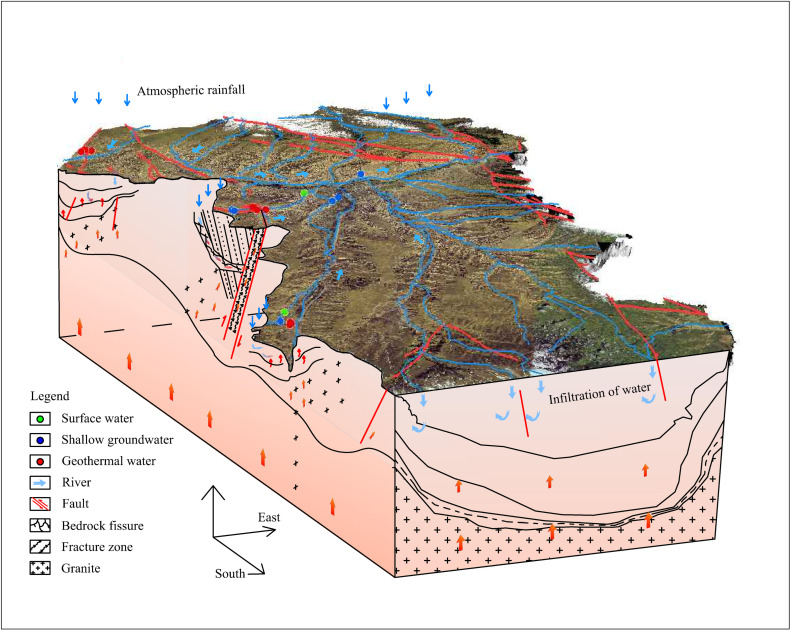
Conceptual model describing the geothermal system in the Guide Basin.

Based on geological, geophysical, geochemical, and hydrological data, we have synthesized the geothermal conceptual model for the Guide Basin (Fig 15). This model primarily illustrates the interaction between heat flow, fluid movement, and recharge mechanisms. Through comparison with known geothermal exploration data, the distribution of heat sources, heat conduction paths, and hydrological flow paths are consistent with actual exploration data, validating the accuracy of the conceptual model.

According to the constructed geothermal conceptual model of the Guide Basin, the geothermal system is divided into the following three parts:

In the Luohantang area (S01–S03), atmospheric precipitation infiltrates along faults and rock fractures, absorbing heat from the slow geothermal flux of granite and the heat generated by the decay of radioactive elements, leading to an increase in temperature. Once the temperature rises to a certain level, the geothermal water begins to ascend through convection, forming a bedrock fracture-type geothermal reservoir along the linearly distributed fractures. In areas where fractures are not well-developed, thermal energy diffuses laterally along the aquifer, creating a layered thermal reservoir. During this process, surrounding rock materials continuously dissolve and filter, mixing with surface water from the Yellow River, resulting in changes to the hydrochemical type of the geothermal water. The geothermal water rises along the fractures at the basin boundary, entering the well-permeable Early Cretaceous sandstone, which is covered by Quaternary sub-sand and sub-clay layers. The Early Cretaceous Miocene consists of mudstone interbedded with fine and coarse sandstones, forming a cap that constitutes the Early Cretaceous thermal reservoir. In summary, the thermal storage in the Luohantang area exhibits characteristics of both layered conductive thermal reservoirs and convective structural thermal reservoirs.

In the Zhacanggou area (S07–S12), near-EW-trending extensional faults serve as pathways for the infiltration of surface water and atmospheric precipitation. The contact metamorphic zone between sandstone and granodiorite acts as a runoff storage and aquifer zone. After infiltration, surface water and atmospheric precipitation continue to absorb heat from the deep crust and from the decay of radioactive elements in crustal rocks during their migration, resulting in increased water temperatures. During the runoff process, the fluid interacts with evaporitic sediments composed of deep-layer rock salt and silicate minerals, leading to high salinity, particularly with the enrichment of Na+ and Cl^−^. When the upward flow of groundwater is obstructed by the overlying Neogene mudstone along the fault, it spills to the surface in the form of upwelling along the structural fault zone, forming a geothermal system characterized by thermal convection mechanisms.

In the Xinjie area (S13–S16), precipitation from high-altitude regions penetrates deep into the strata through faults or fracture zones, absorbing heat from the mantle and the heat generated by the radioactive decay of rocks during subsurface runoff. This results in heated water with temperature variations. During the runoff process, geothermal water flows along specific paths or structures without extensive rock contact for heat transfer, leading to minimal water-rock interaction. When encountering concealed faults, the geothermal water mixes with shallow groundwater, but does not exhibit strong convective movement. This indicates that the geothermal system primarily relies on conduction for heat transfer, categorizing it as a conductive thermal reservoir.

Although the mechanism of geothermal flow mechanics is still speculative to a certain extent, conceptual hydrogeological models are the most reasonable based on the analysis of the hydrogeological environment as well as hydrogeochemical and geothermal measurements.

## 5. Conclusion

This study provides an in-depth analysis of the hydrogeochemical and isotopic characteristics of geothermal water in the Guide Basin, located in the northeastern Tibetan Plateau. It reveals the origin of the geothermal water and the key mechanisms controlling its chemical composition.The main findings are summarized as follows:

**Geothermal Water Types:** The geothermal water types in the Guide Basin exhibit significant variability. The Luohantang and Zhacanggou areas are primarily characterized by Na-SO_4_·Cl type water, while the Xinjie area features Na-HCO_3_ and Na-HCO_3_·Cl type water. The chemical composition of the geothermal water is predominantly influenced by silicate weathering, with carbonate dissolution and cation exchange processes playing a secondary role.

**Isotopic Analysis:** Isotope data (δD, δ^18^O, and ^87^Sr/^86^Sr) indicate that atmospheric precipitation is the primary source of geothermal water in the region. In the Zhacanggou area, geothermal water is primarily influenced by deep water circulation, whereas in the Luohantang and Xinjie areas, there is evidence of mixing with shallower water sources.

**Reservoir Temperature:** The geothermal reservoir temperatures within the Guide Basin range from 82.4 °C to 229 °C, with circulation depths ranging from 2001.6 meters to 58859.5 meters.

These findings provide valuable evidence for the study of the geothermal system in the Guide Basin and serve as a basis for targeted exploration and sustainable utilization of geothermal resources. However, there are also limitations:

The estimation of geothermal reservoir temperatures using the thermometric method is subject to uncertainties due to data limitations and the constraints of the research methodology. These uncertainties may not fully reflect temperature variations in different geological contexts. Additionally, The complexity of the regional geological structure means that certain deep or concealed heat sources may not have been fully identified.

## Supporting information

S1 Table Complete information on the sampled data in this article. (PDF)
