# Supplementary material for: Hydrogeochemical and isotopic analysis for interpreting the formation of the complex geothermal system in the Guide Basin, Northeastern Tibetan Plateau
Source: PLoS One. 2025 Feb 10;20(2):e0317694. doi: 10.1371/journal.pone.0317694 (PMC11809860; doi:10.1371/journal.pone.0317694)
Supplement: S1 Table — (PDF) [file pone.0317694.s001.pdf]

S1 Table. Complete information on the sampled data in this article

Sample number1	Sampling temperature	SiO2	pH	TDS	ES	Na	K	Ca	Mg	Cl	SO4	HCO3	CO3	δD-VSMOW (‰)	δ^{18}O-VSMOW (‰)
**H01**	10	9.68	8	NA	NA	93.63	2.82	98.83	21.34	26.79	194.5	323	0	-66	-9.7
**H02**	12	11.2	8.23	NA	NA	84.85	2.71	60.52	18.23	18.93	173.2	218.4	0	-66	-9.6
**H03**	13	10.92	8.32	NA	NA	134	3.77	86.01	33.03	69.66	245.3	335	0	-56	-8.2
**H04**	11.6	NA	7.24	NA	3520	224.15	9.17	88.2	66.06	273.43	281.29	91.79	0	−67	−9.8
**H05**	10	NA	NA	172	NA	272.2	NA	48.6	34.4	96.3	494.9	219.7	0	-59	-8.6
**H06**	13.9	9.8	7.6	375	764	64.7	4.9	90.4	18.1	48.4	52	307.9	2.1	−57.4	−8.1
**R01**	15.4	NA	8.52	NA	NA	14.85	1.76	56.59	16.3	13.01	41.05	187.3	0	-74	-10.4
**R02**	18	7.8	8.5	183.7	NA	13.7	1.3	53.9	5.8	4.7	12.3	169.8	6.4	−64	−9
**S01**	69.9	163.2	8	1565	3193	695	73.4	47.5	0.4	840.2	266.3	47	0.5	-57	-8.9
**S02**	87	165.2	8	1590	3191	709	73.3	50.4	0.5	844	265	61.9	0.6	−65	−8.7
**S03**	91.9	176.4	8.2	1639	3344	720.5	77	47.4	0.3	891.7	273.5	51.2	1.2	−59	−7.9
**S04**	87	108.3	7.3	1208	2465	403.7	28.5	71.1	0.4	370	630	45.6	3.3	−68	−9.6
**S05**	83.7	111.5	7.3	1259	2568	412.7	29.3	72	0.4	430.9	734.2	46.9	1.4	−69	−10
**S06**	59.7	108.9	8.3	1226	2500	406	31.7	71.9	0.3	409.3	693.5	63.4	3	NA	NA
**S07**	81.9	102.4	8.1	487.8	NA	430	17.6	75.8	0.6	464.7	465.4	65.3	0	-81	-9.7
**S08**	93	119.5	8.4	473.7	NA	443	20	66.3	0.4	471	440.7	26.1	19.3	-82	-10.2
**S09**	52.7	100.2	8.6	476.6	NA	441	19.7	65.4	0.2	4448.7	469	26.1	19.3	-83	-10.2
**S10**	67.2	NA	7.88	NA	2580	423.72	20.2	56.4	0.28	356.18	518.61	62.77	0	−84	−10.8
**S11**	90.2	NA	8.37	NA	2530	389.82	17.14	63.01	3.94	334.26	532.54	55.79	0	−85	−11
**S12**	80	NA	8.5	NA	2700	305.08	19.18	85.8	3.8	337.68	386.43	80.8	0	−84	−10.9
**S13**	74	68.9	8.5	504.6	NA	156	5.5	10	2.4	28.4	235.4	109.8	12	-94	-12.8
**S14**	48.9	71	8.3	488	995	235	12.4	12.8	0.3	43.8	257.9	116.2	1.5	−93.2	−12.3
**S15**	66	75.8	8.4	484	996	227	10.4	16.1	0.4	43.4	246.3	122.1	1	−91.5	−12.3
**S16**	52.5	75.3	8.3	481	981	220.8	10.6	19.5	0.8	41.6	240.6	131	0.7	−90.6	−12.1
